# Improving the quality of life of palliative and chronic disease patients and carers in remote Australia with the establishment of a day respite facility

**DOI:** 10.1186/s12904-016-0136-1

**Published:** 2016-07-18

**Authors:** Timothy A. Carey, Kellie Schouten, John Wakerman, John S. Humphreys, Fred Miegel, Simon Murphy, Mick Arundell

**Affiliations:** Centre for Remote Health, PO Box 4066, Alice Springs, Northern Territory 0871 Australia; Monash University School of Rural Health, Melbourne, Australia; Northern Territory Department of Health, Darwin, Australia

## Abstract

**Background:**

In the Northern Territory (NT) there is a lack of respite services available to palliative care patients and their families. Indigenous people in the NT suffer substantially higher rates of poorly controlled chronic disease and premature mortality associated with poor heath than the Australian population as a whole. The need for a flexible, community based, culturally appropriate respite service in Alice Springs was identified and, after the service had been operating for 10 months, a qualitative evaluation was conducted to investigate the experiences of people involved in the use and operation of the service.

**Methods:**

Semi-structured interviews were conducted with patients, carers, referrers, and stakeholders. A total of 20 people were interviewed. Interpretative Phenomenological Analysis was used inductively to analyse the transcripts. Two case studies are also described which illustrate in greater detail the impact the respite service has had on people’s lives.

**Results:**

From the semi-structured interviews, two superordinate themes along with a number of sub themes were developed. The two superordinate themes described both “The Big Picture” considerations as well as the pragmatics of “Making the Service Work”. The sub themes highlighted issues such as being stuck at home and the relief that respite provided. The case studies poignantly illustrate the difference the respite service made to the quality of life of two patients.

**Discussion:**

The findings clearly indicate an improvement in quality of life for respite patients and their carers. The respite service enabled improved care coordination of chronic and complex patients as well as improved medication compliance and symptom management. As a result of this evaluation a number of recommendations to continue and improve the service are provided.

## Background

Palliative care services for people facing life threatening illnesses are important for improving pain management, life expectancy, and symptom control of illnesses [1, 2]. While not all people facing life threatening illnesses need specialist palliative care, some population groups are more vulnerable and have greater unmet needs than other groups. These groups include: Aboriginal and Torres Strait Islander people; culturally and linguistically diverse populations; rural and remote populations; and socially and financially disadvantaged populations [3].

### Palliative care, rurality, and indigenous health

In the Northern Territory (NT), usage patterns of palliative care services differ somewhat to other Australian jurisdictions due to the high Indigenous population who have poorer health status and higher rates of premature mortality and chronic disease. Chronic disease, for example, constitutes a larger proportion of the palliative care workload than cancer in the NT [4]. The difference in disease profile between the NT and the rest of Australia emphasises the need for flexible, community based palliative care services to maintain symptom control associated with disease and thus prevent avoidable hospitalisations associated with poor disease management. The need for such a flexible model of service delivery is further emphasized by the particular geography of the NT, with its large distances between small isolated communities and harsh climate that often makes transport extremely difficult or impossible.

Across rural and remote Australia, including the NT, there is a lack of respite services available for palliative care patients and families and a lack of respite services culturally specific to Indigenous people [5]. Caring for dying people can be an overwhelming task [5]. Indigenous people are heavily burdened by carer responsibility which is compounded by poverty and a serious lack of resources [5]. Researchers found that hospital was the only place available for palliative care respite in the NT outside of Darwin, but this is considered to be ad hoc, informal, or short term, and causes upheaval to patients and families [5]. This lack of respite care obstructs patients’ and carers’ wishes for death to occur in the local community.

At the present time only 16 % of the Australian population die at home with the remaining 84 % dying in institutions [3]. Most people when asked, however, would prefer to die at home [3]. Particularly for Indigenous people, given their strong connections to the land and their family, the need to relocate for respite can create significant psychological turmoil [5]. Furthermore, hospital deaths also result in a potentially poorer quality of death [6].

### Respite care

Respite care, the temporary provision of institutional care for the relief of carers, can be the cornerstone to providing care to many vulnerable populations, including children, elderly, and dying people. It is provided in the forms of day care, in home respite care, or night care [7]. Respite facilities have a role in supporting people with their wishes to die at home by providing community based, professional, palliative care support. Indigenous Australians, however, are particularly disadvantaged in terms of the provision of respite services [5].

Qualitative studies of respite services have found numerous benefits to patients and their carers. Palliative day care increases feelings of psychological wellbeing, self-worth and self-esteem, reduces social isolation for dying people, and helps to normalise suffering. Palliated people often feel “temporally and socially bounded”, and attending day care distracts them from feelings of illness as well as providing peer support for the individual and respite for carers [8–11]. One study in Australia found that palliative day care was the preferred type of respite care for carers [12].

### The current study

In 2010 Alice Springs successfully gained funds to purchase, refurbish, and operate a house in the community. There was also a commitment to evaluate the impact of the service after the first 10 months of operation. Broadly, the research question being investigated was: “What is the personal impact on patients and carers of the respite service?”.

## Methods

### Design

The design of the research was a cross-sectional qualitative study. The research assistant (RA) who conducted the interviews was a non-Indigenous allied health professional who was experienced in visiting remote communities and working with Indigenous people from a health service provider perspective.

Semi-structured interviews were conducted individually. Patients, carers, referrers, and stakeholders were interviewed separately to identify barriers and facilitators to this service delivery model. Topic guides (see Appendix) covering people’s experiences of respite care as well as barriers and enablers to using the facility were used to provide a loose structure for the interviews. Interviews started broadly with a question such as “What has been your experience of respite care?”. All interviews were audio recorded, transcribed into Microsoft word, and each transcript was read and key ideas and themes were identified initially by the RA. Then, themes between participants were integrated and synthesised. The themes were discussed with the primary researcher and transcripts were revisited when doubts or uncertainty about the themes arose.

Two participants were purposively selected as case studies for the project because of the significant impact the respite facility had for them. These participants were interviewed but, in addition to their interview data, information regarding hospital admissions was also investigated. Their interview data were included and analysed with the data from the other participants but their hospital admissions data are presented separately in the case studies.

### Participants

The groups from which people were recruited (see Table 1) were: Stakeholders-people who had been involved in the initiation and establishment of the program; Referrers-people who referred other people to the program; Patients-people who attended the respite service and participated in various program activities; and, Carers-people who were carers of patients who had participated in various activities of the program. An invitation to participate in the study was extended to all those who were involved in the operation or uptake of the service. During the 10 month period of the evaluation, 23 people were referred to the service. From these 23 referrals, 17 people used the service and 13 people used the service on a regular basis. A small number of patients were referred to the service but their referrals were unable to be accepted for reasons such as patients requiring one-to-one care, patient load, and staff capacity. Five of these 13 people and their carers agreed to participate in the study. Of the five stakeholders, three agreed to participate in the study and all seven of the referrers consented to participate.

**Table 1:** Themes identified in the analysis of interviews

**Superordinate Themes**	The Big Picture	Making the Service Work
**Sub Themes**	• Respite shortage in Alice Springs	• Service operation
	• Stuck at home	• Activities at the house
	• Respite provides relief	• Spirit of the respite service

The experiences of two regular patients of the day respite service are described in some detail as case studies to highlight the individual benefits that can often be obscured when only group data are considered. The impact that attending the respite facility had on the lives of these two men is instructive.

### Setting

The Alice Springs Palliative Care Service of the Northern Territory Department of Health is a consultancy service that covers the Southern Region of the Northern Territory into the Anangu Pitjantjatjara Yankunytjatjara (APY) Lands of South Australia and the Ngaanyatjarra lands of Western Australia. The Service provides support, advice, and education to primary care providers regarding end of life care. The Service consults in urban institutions (Alice Springs and Tennant Creek Hospitals and aged care facilities), urban primary care settings (GP practices, Aboriginal health services), and in remote settings (remote clinics and aged care facilities).

Staff of the Palliative Care Service, including the Respite House, have developed key networks with local Aboriginal health and wellbeing service providers who assist in coordinating care of the clients. These networks have been established by providing transport as well as by primary health care staff visiting the clients within the Respite house. For example the Frail Aged and Disabled (FAAD) team from Central Australian Aboriginal Congress (CAAC) Health service, coordinate their reviews of patients with the Respite House staff.

Alice Springs has a population of 28,500 with 20 % of the population identifying as Aboriginal [13]. The lifestyle of the Aboriginal population is variable within the township. Very urbanised Aboriginal people live in mainstream housing. It is estimated that 3300 or 58 % of Aboriginal people within Alice Springs live in “town camps” [14] which were originally designated as reserves on the outskirts of the township but, in many cases, have now been engulfed into the township as the town has grown. Many of the residents of town camps have transient families from remote settings who visit Alice Springs for the purposes of shopping, health needs, and legal matters compounding issues of overcrowding. There is a small population of Aboriginal people in Alice Springs who are homeless. These people stay where they can which often includes family members living in town.

### Procedure

Initially, the RA spent time visiting the respite facility during the initial months of operation to engage with patients and understand the functioning of the centre. The RA provided information about the project as well as consent forms to stakeholders, referrers, patients, and carers. By recruiting people from different groups to the study it was intended that the results would be triangulated to converge on a more in-depth understanding of the way in which the facility was experienced. People who consented to participate in the study were contacted to arrange an individual interview with the RA at a mutually convenient time and location. Interviews typically lasted between 30 and 60 min. Interviews were audiotaped and transcribed and participants were provided with the opportunity to review the transcripts prior to analysis.

### Analysis

Interpretative Phenomenological Analysis (IPA) [15] was used to analyse interview transcripts. IPA is an iterative, inductive process of constructing superordinate themes and sub themes where transcripts are constantly revisited to ensure that the superordinate themes directly relate to the shared experiences of the participants. Since IPA is focussed on exploring the lived experience of participants from their own unique perspectives and of developing shared understandings of these experiences it was considered an ideal form of analysis for this research. Initially, transcripts were read multiple times and words or phrases that appeared to address the research question were highlighted. Next, highlighted words and phrases were collated into tables with each participant’s comments occupying one column and different tables developed for each participant group. Within these tables individual participant themes were identified and then synthesised across participants and across tables to develop a coherent summary of the interviews. Finally, the themes that were developed from the transcripts were grouped according to common concepts to form superordinate themes and sub themes.

## Results

From the analysis of the data, two superordinate themes and six sub themes were developed (see Table 1). The superordinate theme “The Big Picture” covered broad issues such as the availability of respite in Alice Springs, carer burden resulting from a respite shortage, and external factors affecting the establishment of the program and impacting on patients and carers. Many of these issues crossover with the issue of remoteness. “Making the Service Work” covers organisational considerations involved in the establishment and ongoing operation of the program that affected the experience of patients and carers.

### The big picture

#### Respite shortage in Alice Springs

This theme emerged through a strong consensus among carers, stakeholders, and referrers that there are very few options for respite in Alice Springs. As the care needs of individuals increased, the need for respite increased, but options reduced. Participants spoke of the general lack of options for respite in Alice Springs. Carers of patients with high and increasing care needs spoke of being overburdened with no simple option for respite. Available respite in Alice Springs generally involves being placed on long waiting lists. Obtaining respite for high care patients is difficult and at times impossible. Referrers found that patients were misusing services such as Alice Springs Hospital in order to have basic needs or primary health care needs met, and the lack of options for these people perpetuated this cycle.

*… there have been less and less options for him, so to have this at a point where his care needs have never been so high is incredible* (C1)

Referrers and stakeholders spoke of patients with chronic and complex conditions who are not strictly palliative, not fitting into the criteria for many services, and “slipping through the cracks”. Consequently the health status and symptom management of these people was poor. Marginalised patients such as those living in town camps or Indigenous people generally who were requiring the respite service tended to be placed in situations of risk when no option for respite was available including being left at home alone. Moreover, carers often had difficulty meeting the basic needs of patients due to issues relating to poverty such as transport, overcrowding, low income, and poor hygiene. The introduction of the respite service meant that these patients now have a safe place to go during the day, they can have their health care and personal care needs met, and services have a point of contact to assist with coordinating patients’ health care needs.

#### Stuck at home

Overwhelmingly, participants discussed the issues of both carers being housebound due to their caring responsibilities and patients being housebound due to a lack of suitable services available in Alice Springs to provide relief. Many emphasised the burden experienced by carers who had loved ones with high care needs. In most cases high care patients were difficult to transport and therefore carers required support to do their jobs outside of the home. Issues of isolation, quality of life, and social and emotional wellbeing were all identified as problems for both carers and patients as a result of being “stuck at home”.

*I couldn’t take him anywhere, we used to up until Christmas … I used to teach night classes and I used to bring him, I actually enrolled him, he didn’t do anything he wasn’t able to, I needed to have him there because I didn’t have anyone to care for him* (C1)

*… it’s a never ending workforce, the only time we can relax is when she’s asleep* (C2)

Patients commented that, if the respite service was not available, they would be sitting at home on their own. Indigenous patients from town camps were more likely to report this.

*I’d just be sittin’ down at home* (P2)

*… no-one would be at home, my wife would be working … she worry, that’s why she wanted to find a place where I can stay for the day* (P2)

Carers reported that as their loved ones’ care needs increased, their options for respite declined.

*As his mobility has deteriorated, the continence issues, the um swallowing stuff, because now he’s on the highest grade, all things are thickened into pudding thickness. Um so all of this has happened now there have been less and less options for him* (C1)

#### Respite provides relief

Generally, participants expressed satisfaction and relief that the service was now available. Referrers were strongly encouraging of the service including the quality of the service and the facility as well as the flexibility of the staff to meet the needs of complex patients. Carers reported that the introduction of the respite service gave them an option for respite, where their options for respite were declining due to their loved ones’ increasing care needs. Exhaustion, isolation, loneliness, and anxiety were identified as issues for carers and patients before the introduction of the respite house.

*I wasn’t getting enough of a break, and there just didn’t seem to be any way to get a break, it was just putting one foot in front of the other, he was housebound, I was largely housebound, um, yeah, if I did get a break, I was often so tired that I’d just go into my room and sleep, so yeah, although I was very grateful for it, it wasn’t enough* (C1)

*I always think that when he’s home (alone) I always think he might fall down or walk the wrong way* (C3)

The introduction of the respite house has improved the quality of life and emotional wellbeing among carers and patients.

*It’s given me a life back … Well I feel a lot freer, a lot more rested, I have more options, more time to do stuff, um as we are getting more into the routine of it, I guess I’m counting on it more* (C1)

*It makes me happy that he’s gone there, so I can have time to do some sewing and things for me you know* (C3)

*I’ve used up all my holidays, all my long service, all my sick, I’m looking after her three days per week, then I can only work two days per week. Now I have no back up at all as far as long service or holidays go, that’s all gone, and it was just ideal that [location] came up at the right time, yeah, it’s really excellent* (C2)

*All I can say is, I love coming here* (P2)

Referrers reported struggling to coordinate marginalised patients with high care needs who misused services and had poorly managed chronic diseases. With the introduction of the respite service this has provided a place for complex patients to attend, have their medical and basic needs met, and at times, therapeutic needs also. Referrers reported that symptom management, medication compliance, quality of life, and service coordination had all improved for these patients, as well as an improvement in the referrers’ ability to manage these patients. The provision of a “safe place” for isolated and marginalised members of the community suffering from complex conditions was strongly emphasised as an important outcome.

*[Before] he would call me four to five days of the week and he would need things like, to get to [Medical Service], his meds had run out, he needed his pain patch changed, when he was feeling unwell he would present to the emergency department at ASH at least twice per week he would be admitted and it would usually be pain related* (R2)

*[After] he just took to it like a duck to water really and it has just really improved the quality of his life dramatically, I think he really feels like he’s got a place there and a great purpose … to actually have somewhere where he can feel he has a meaningful role has been important for his self esteem* (R2)

*The benefits were enormous, he was very disengaged from the health care system when I first became involved with him, he was using the emergency department completely inappropriately, he was presenting on average three or four times per week for dressing changes and would be quite sick and get admitted and then two and three days later take his own leave before his health issues were addressed and it was a really unhealthy cycle of dependence on the hospital without actually utilising the services properly. He then was supposed to be seen by the community nurses and would often not be home when he was supposed to be seen by the community nurses or they would get there and he would tell them to go away, so they were changing his dressing irregularly so his wounds were deteriorating and he had a maggot infestation and so it was really not working well. Once we got him into a routine of going to the house three days a week it worked out really nicely. The community nurses could go to the house, they would know he would be there, he could have a shower before they’d do the dressings so was optimal conditions and once he started going to the house regularly his presentations to the emergency department dropped off so significantly, so much that he had three presentations over an eight week period, compared to three times per week before that* (R1)

### Making the service work

#### Service operation

Participants described factors that strongly influenced the day-to-day operation of the service. Many participants identified staffing as a strong contributor to the success of the program and as an important factor in the consistency of the program. Many identified that those trust relationships that were built between patients and the respite staff were an integral part of successfully engaging people with the program. Some patients felt as though they had built genuine friendships with the staff at the house.

*Can I just say [staff member] and [staff member] are my friends* (P1)

Problems with staffing were identified throughout the interviews as a factor that affected the consistency of the program and impacted on the service provided to patients and carers.

*I think the hurdles for running the house is looking for the right people to work in there, and that’s an interesting process in itself, and you’re always going to have those problems in Alice Springs, of getting the right personalities. I know programs that have fallen over because certain people have left and the passion has left with them* (S1)

*… some problems we’ve been dealing with, consistent staff, you know, sick leave, you know these things happen, you know dealing with that and putting in that back up plan* (S1)

#### Activities at the house

Participants discussed the service provided by the respite house as well as identifying things that were needed or missing. The respite house provides a service that includes day respite, transport to and from the house, provision of meals and hydration, medical needs such as wound dressings, oxygen, and management of medication. Personal care needs such as showering and washing clothes and changing pads are also provided. The provision of medical support and having a Registered Nurse employed were factors that gave carers, referrers, and patients confidence in using or attending the service.

*He has on occasions needed a nebuliser, but because they have an RN on staff there they can just assess that and say right we’re gunna do this now. With his breakfast he has morning medications and they crush it and give it to him* (C1)

*I was completely comfortable when you know that there’s a qualified nurse there and friendly [staff member] making scones and pikelets, they make us feel really comfortable* (C4)

Staff provided transport for patients to and from the house. This was identified as an important feature of the service as transport for many was identified as a barrier to attending the service. Provision of transport by other services and the taxi company were considered unreliable, and the success of getting people who did not have transport to the program, depended on the availability of the respite service to provide this transport. Transport of mobility impaired or wheelchair bound patients was not possible as the vehicle used by the respite service is not accessible for these patients, there are few wheelchair taxis available in Alice Springs, and other agencies with these facilities were not dropping off and picking up patients in time. The addition of a wheelchair bus was strongly recommended throughout the interviews. Indigenous people or those living on town camps were more likely to be dependent on transport.

Patients described their time at the house as relaxed and many simply enjoyed going to the house and sitting around, sleeping, or watching television.

*When I’m sitting at home, sometimes my family they go into town, they do shopping and they leave me alone, I mean, I’m on my own, and since I came here these other people from other town camps, I know we don’t talk much, but we get together and just sit around and watch TV … These people that work here they getting to know us so we like to make sometime tell story to one another, or sometime make joke of one another, so I’m happy yeah* (P2)

Some participants indicated that a schedule of activities should be created to enhance the service. Interestingly, this initiative was mainly shared by stakeholders and referrers and was not raised by patients. Patients were satisfied with the basic service that was provided.

#### Spirit of the service

There was an emphatic view that this service was unique in its delivery and that the staff had a strong “can do” attitude, and tended to go to great lengths to meet the needs of patients. Many felt that the service was tailor-made to the particular needs of patients, and there was a strong sense of motivation, persistence, and enthusiasm from staff, to overcome barriers for patients to get to the service.

*I am just amazed that I went through all of [patient’s] care needs with [staff member] and he was not phased at all … I think we might have a solution* (C1)

The service was described by many as flexible and accommodating. Referrers felt as though they could refer a wide range of patients who were not suitable for other services and the respite service would at least consider them.

### Case studies

Two case studies have been included as supplements to the other qualitative data to provide a more fine-grained illustration of the impact of the respite service for people who used the service regularly. Two male participants were chosen as case studies because of the complexity of their situations as well as their different experiences engaging with the facility. One participant engaged readily with the program whereas the other participant was more reluctant to engage initially.

#### Case study one

Rob (pseudonym) is a 62 year old Indigenous man living on a town camp in Alice Springs. Rob suffers from a slowly progressing carcinoma with bone metastases. He is originally from a community south of Alice Springs. Prior to receiving his diagnosis he was a strong working man, however, following his diagnosis he was told he was not allowed to work. Rob lives alone and his family occasionally visit. Rob is under the care of the palliative care team.

Prior to the commencement of the respite service Rob contacted the palliative care team four to five days per week for symptom related issues. He also called an ambulance on a weekly basis for pain related issues. In the 12 months prior to the commencement of the respite service Rob had 35 attendances to the emergency department (ED), four hospital admissions, and stayed 66 days in hospital. By visiting Rob in hospital, the palliative care team found that his admissions were largely due to pain. Rob was generally isolated and would often present to the hospital just to “chat” to staff.

Following the opening of the respite service, Rob started attending on a regular basis. The respite service provided Rob transport to the house, meals, and assisted with the management of his medications. They were able to monitor any symptom related issues and coordinate care for Rob. When Rob attended the facility he would take care of the garden and assist the staff by helping to communicate the needs of other patients. He felt as though he had a genuine role in the house. In this period his ED attendances dropped to five (from 35) and he had no hospital admissions.

Attendance at the respite service reduced Rob's isolation, it provided him with connection, reduced his anxiety around his condition, and improved symptom management as well as access to primary health care support.

#### Case study two

Jimmy (pseudonym) is a 47 year old, homeless, Indigenous man, suffering from end stage bronchiectasis and is dependent on home oxygen. Jimmy is under the care of the palliative care team as well as other primary health care programs. Agencies involved in the care of Jimmy had problems with his care coordination as he was difficult to locate and did not present for medications or appointments. For the 12 months prior to the commencement of the respite service, Jimmy presented to the emergency department on a nightly basis for a bed to sleep in and had 74 ED presentations and 930.9 ED hours.

Initially, Jimmy was reluctant to attend the respite service, but since it opened he has attended between 3 to 5 days per week. This has enabled improved service coordination for Jimmy, and agencies involved in his care have been able to assist him with his homelessness issues. Because Jimmy’s homelessness issues have not resolved, there has not been any improvement in his ED presentations.

In the period after the respite service opened he presented to ED on 119 occasions for a total of 1841.9 h. Although Jimmy’s presentations to ED increased, his number of admissions to hospital from ED reduced from 32 to 7 after the respite service opened. Despite the increase in the usage of ED due to his issue of homelessness there was less medical intervention needed resulting in a cost saving of $50,239.56 for this patient. This suggests there may have been some improvement in Jimmy’s symptom management and that his admissions to the ward were as a result of genuine exacerbations of his illness rather than poor symptom management.

Since the commencement of the respite service, Jimmy now has his medications daily, his symptoms have improved, and during the day he has a bed to sleep in. For a period he did access hostel accommodation and, during this period, his ED presentations stopped completely. He is still homeless but now that the respite service is available to him, he sleeps in ED overnight and contacts the respite service in the morning to be picked up. Thus, Jimmy’s involvement with the respite service has improved his isolation, improved other agencies’ abilities to support him, and improved his symptom management.

## Discussion

The impact of the community respite service has been strongly positive which indicates it has been an appropriate response to an identified need in the Alice Springs Community. Palliative care and respite services in central Australia are vastly complex, with issues of remoteness, a high proportion of Indigenous people in the population, high levels of chronic diseases, and high levels of poor living conditions needing to be factored into the development of a service [4]. The findings of this evaluation indicate that providing a day respite facility for palliative and chronic disease patients was beneficial for both the patients and their carers.

It appeared to be the case that the willingness of the staff to work flexibly and responsively ensured that marginalised people were able to access the service in culturally appropriate ways and this seemed to be an important factor in the positive impact of the service. Patients were able to access the service when they needed to rather than attending a prescribed program and staff spent time understanding the individual requirements, including the cultural considerations, of each patient. The facility was staffed by experienced health professionals who took the time to enquire about the perspectives of the individual patients and carers and they used these perspectives to inform their practice. The respite centre filled a gap in services for those marginalised Indigenous people suffering from chronic and complex conditions who are not yet in need of intensive end of life care. In the absence of such a service these patients were often isolated, living alone, marginalised, and not accessing services either available to them or appropriately. Often their symptoms were poorly managed and their enjoyment of daily living was greatly reduced. The necessity of flexibility and cultural appropriateness in end of life care was emphasised fifteen years ago [16] but these important considerations are still not realised on a widespread basis.

Additionally, referrers stated that the introduction of the respite house had made a tangible difference to their workload as their time spent with complex patients had reduced. Carers overwhelmingly reported that it gave them a much needed break, reduced the pressure in their lives, and allowed them to be more productive. These reports are consistent with information found in the literature indicating that respite services for Indigenous people and their carers are in shortage and desperately needed [5].

### Limitations

One of the limitations of the study concerned the time frame within which the study was conducted. External considerations required that the program was evaluated after ten months of operation. It is possible that different information may have been obtained had the facility been evaluated after eighteen months or two years. Despite this limitation the experiences of people participating in the evaluation were overwhelmingly positive even in situations where they might have been initially reluctant to engage.

Another limitation of the project may be the question of transferability of the findings. Given the unique features of this facility in the context of remote Australia there could be doubts regarding the extent to which these findings would transfer to other settings. One of the lessons from this evaluation, however, was the demonstration of important principles of practice such as flexibility and cultural appropriateness. It is these principles which would be transferable to other contexts even though the particular programs may be different across different locations.

### Future research

The establishment of facilities such as the one described in this study would benefit from longer term research. Indeed, a research and evaluation component should be an aspect of ongoing funding for the facility. Research that occurred over an extended time frame would be able to provide a more complete understanding of the support needs of palliative and chronic disease patients and their carers in this complex remote context. There would also be the opportunity to design and test different supportive programs. Furthermore, an extended program of evaluation would enable a more fine-grained analysis of the factors affecting the positive impact of the service. What are the important characteristics that enable staff to work flexibly and responsively? How are cultural considerations most effectively accommodated? How important is strong leadership and staff training in the impact of the service?

The respite facility could be extended in different ways depending on the funding available and this would provide scope for further research. Extending the facility to include overnight accommodation would address some issues of homelessness and would provide extra respite resources for carers. Having other options like overnight accommodation in place would provide additional avenues for research to better understand the palliative and chronic disease experience in remote settings and how best to support it.

## Conclusions

It seems clear from this qualitative evaluation that the establishment of the respite service has met an important unmet need in the Alice Springs region. The qualitative findings and case studies clearly indicate that the establishment of the respite service has resulted in marked improvements in daily living for respite patients and their carers. Establishing the respite service improved case management of chronic and complex patients. Over time, the service became a “hub” for chronic and complex patients. The provision of regular support with medicines, wound care, and other health care needs helped to stabilise people and prevent acute exacerbations of illness.

Extending and improving the service could occur in a variety of ways. Ensuring that appropriately trained staff are recruited and that a mix of both male and female staff are included is important. Providing reliable transport facilities would assist patients in attending the service on a regular basis. Exploring options to cater for overnight attendances at the facility was seen as a desirable initiative for the future.

The respite facility for palliative and chronic disease patients was seen as a flexible, culturally appropriate, and effective response to an urgent community need. Continuation and expansion of this resource would help improve day-to-day living for palliative and chronic disease patients and their carers in remote Australia.

## Appendix

**Topic Guides**

Evaluation of the Palliative Care day respite centre

*Respite Participants*

1. Before the respite centre opened
  - What was your experience of respite care?
  - What sort of support did you receive from the palliative care team?
  - How satisfied were you with palliative care services before the respite centre opened?
2. Experience of respite centre
  - What’s been your experience of the respite centre?
  - How satisfied are you with the new respite service?
  - What changes have occurred since attending the respite centre?
3. Barriers and enablers to attending the respite centre
  - Are there any barriers to attending the respite facility?
  - Is transportation an issue?
  - Is family support an issue?
  - Is accommodation an issue?
  - Others?

*Carers/family*

1. Before the respite centre opened
  - Did you receive respite services before the centre opened?
  - What sort of support did you receive from the palliative care team?
  - How satisfied were you with palliative care services before the respite centre opened?
2. Experience of respite centre
  - What care/support does your dependent receive at the respite centre?
  - How satisfied are you with the new respite service?
  - What changes have occurred since you have been utilizing the respite service?
3. Barriers and enablers to attending the respite centre
  - Are there any barriers to attending the respite facility?
  - Is transportation an issue?
  - Is family support an issue?
  - Is accommodation an issue?
  - Others?

*Stakeholders*

1. Establishment of the respite house
  - Motivation?
  - Hurdles, problems, barriers?
  - How were they overcome?
2. Progress of the respite house
  - What were things like initially?
  - What are they like now?
  - Pace of progress?
  - Tracking of progress, evaluation procedures, accountability?
3. Benefits of the respite house
  - What has been noticed?
  - Who has noticed it?
  - Has it been worthwhile?
4. Future ways of working and activities for respite house
  - What next?
  - What would you recommend to other communities?

*Referrers*

1. Knowledge of what the respite house does
  - How did you learn about it?
  - What do you know of it?
  - Has your knowledge of the respite centre changed over time? In what ways?
2. Use of the Respite service
  - How often do you refer to it?
  - What clients do you refer?
  - Are there some clients you wouldn’t refer to the centre?
3. Perspective of the Respite service
  - What has your experience of the respite service been?
  - What benefits have you noticed?
  - Could anything be different?
4. Advice for the respite team?
  - Recommendations?
